# Value conflicts in mothers' snack choice for their 2‐ to 7‐year‐old children

**DOI:** 10.1111/mcn.12860

**Published:** 2019-07-10

**Authors:** Femke W.M. Damen, Pieternel A. Luning, Gert Jan Hofstede, Vincenzo Fogliano, Bea L.P.A. Steenbekkers

**Affiliations:** ^1^ Food Quality and Design Group, Department of Agrotechnology and Food Sciences Wageningen University & Research Wageningen The Netherlands; ^2^ Information Technology Group, Department of Social Sciences Wageningen University & Research The Netherlands; ^3^ North‐West University South Africa

**Keywords:** children's dietary behaviour, diary research, food choice, healthy snack, interview, value conflict

## Abstract

Value conflicts appear when people experience struggles, doubts, and feelings of guilt when making food choices. This study aims to provide insight into value conflicts, which mothers may experience while providing snacks to their young children. Mothers are mainly responsible for providing the snacks their young children eat, making it a big responsibility for them as children's dietary behaviour tracks into adulthood. Possible value conflicts Dutch mothers (*n* = 136) experience while providing snacks to their 2‐ to 7‐year‐old children were investigated using food and motivation diaries and semi‐structured interviews. Differences between mothers' educational level, first versus not‐first child, and the differences in age of the children were taken into account. Results showed that the younger the children, the more value conflicts the mothers experienced. Mothers experienced most value conflicts when they provided snacks perceived as unhealthy. Six main value conflicts are elicited by this study, namely, conflicts between healthy and unhealthy snacks; conflicts between healthy and convenient snacks; conflicts related to providing snacks just before dinner; conflicts related to influence of others; conflicts when the child asks but the mother says “no”; and conflicts related to many unhealthy snacks at parties or visits. The insights gained in this study can be used for interventions to promote a healthier lifestyle, support the design of new snack products, and can give guidance for marketing challenges in global snack markets.

**Key messages**

- The younger the children, the more value conflicts the mothers experienced while providing snacks.
- Mothers experienced most value conflicts when they provided snacks perceived as unhealthy.
- Six main value conflicts are elicited, that is, conflicts between healthy and unhealthy snacks; conflicts between healthy and convenient snacks; conflicts related to providing snacks just before dinner; conflicts related to influence of others; conflicts when the child asks but the mother says “no”; and conflicts related to many unhealthy snacks at parties or visits.

## 1 INTRODUCTION

Food choice is one of the most frequent human behaviours. Many factors and their interactions determine the complexity of this behaviour (Köster, 2009). When children are young, their parents are mainly responsible for providing the foods their children eat (Boots, Tiggemann, Corsini, & Mattiske, 2015; Hennessy, Hughes, Goldberg, Hyatt, & Economos, 2012). This makes it a big responsibility for parents as children's dietary behaviour tracks into adulthood (Craigie, Lake, Kelly, Adamson, & Mathers, 2011; Nicklaus, 2016). A considerable part of this dietary behaviour is the frequent intake of energy‐dense snacks by children (Boots et al., 2015; Gevers, Kremers, de Vries, & van Assema, 2016; Piernas & Popkin, 2010). In the Netherlands, snack consumption among children is highly prevalent too; according to the Dutch National Food Consumption Survey of young children, aged 2–6 years, 77% of the children has three or more eating occasions a day, besides the main meals (Ocké et al., 2008). This makes snacking an important factor contributing to childhood overweight (Larson & Story, 2013). Childhood overweight is a serious problem as it increases the risk of health problems, not only in childhood but also later in life (Daniels, 2009; Reilly & Kelly, 2011).

Consumers' food choices depend on the consumer's value system (Osinga & Hofstede, 2004), as consumers associate a variety of values with food (Luomala, Laaksonen, & Leipamaa, 2004). According to the food choice model of Furst, Connors, Bisogni, Sobal, and Falk (1996), major choice‐related values are taste, cost, convenience, health and nutrition, interpersonal interactions, and quality. Safety is also an important food choice‐related value (Lusk & Briggeman, 2009). These multiple food‐related values can cause value conflicts, as they do not always serve the same purpose (Furst et al., 1996). Value conflicts happen when fulfilling one value prevents meeting another value (Connors, Bisogni, Sobal, & Devine, 2001). Luomala et al. (2004) investigated value conflicts that consumers may experience while making food choices. Conflicts between convenience and care and between health and indulgence appeared to be major food‐related value conflicts.

Mothers are of particular importance in food and snack choice for their children (Holsten, Deatrick, Kumanyika, Pinto‐Martin, & Compher, 2012; Walsh, Meagher‐Stewart, & Macdonald, 2015) as they are mainly responsible for food provision (Hardcastle & Blake, 2016; Jones, 2018). Value conflicts may appear when mothers experience struggles, doubts, and feelings of guilt when making food choices for their children. In a study by Hayter et al. (2015), parents mentioned differences between what they would like to feed their children and what they actually provide. Others found that the more unhealthy the food, the more feelings of guilt mothers experienced (Pescud & Pettigrew, 2014). Likewise, Okada (2005) concluded that people experience a higher need for justifying a hedonic choice compared with a healthier choice. Bahr‐Bugge and Almås (2006) found that when Norwegian women served pizza, they always defended and justified this unhealthy and convenient choice.

Usually, mothers would like to make the best choices for their child, and therefore, they could experience difficulties and feelings of doubt (Fielding‐Singh, 2017; Gram, Hohnen, & Pedersen, 2017; Johnson, Sharkey, Dean, Alex McIntosh, & Kubena, 2011; Machín, Giménez, Curutchet, Martínez, & Ares, 2016). In fact, mothers wish to combine healthy food choices, with their child's taste preferences (Boak et al., 2016; Carnell, Cooke, Cheng, Robbins, & Wardle, 2011; Damen, Luning, Fogliano, & Steenbekkers, 2019; Walsh et al., 2015; Wijtzes et al., 2017), two values that often conflict (Luomala et al., 2004). To which extent value conflicts occur in snack choice has not yet been studied. This study aims to provide insight into value conflicts mothers may experience while providing a snack to their young children, using analysis of diaries and semi‐structured interviews. Differences between mothers' educational level, first versus not‐first child, and the differences in age of the children were also taken into account.

## 2 METHOD

### 2.1 Study design

Possible value conflicts Dutch mothers experience while providing snacks to their 2‐ to 7‐year‐old children were investigated. Value conflicts include, in this study, both conflicts between two separate values (e.g., health vs convenience) and interpersonal activity conflicts (e.g., conflicts related to influence of others). The age range of 2–7 years was set, according to the age ranges of Piaget and Inhelder (2000). They developed a model with four consecutive stages of age groups in which the information processing capacities of children increase, their thinking changes from concrete to more logical and abstract, and problem solving and reasoning skills become more advanced. Furthermore, according to Contento (1981), who studied how children think about food and eating, children in the preoperational stage (2–7 year old) do not make the distinction between foods and snacks. This is of importance for this research, because it shows that it does not make sense to ask young children themselves about their food choice during snacking moments but to ask their mothers. Therefore, mothers were chosen as the target group in our study, because children in the range of 2–7 years usually do not pick snacks themselves but receive it from their caregivers (Jacquier, Gatrell, & Bingley, 2017; Ventura & Worobey, 2013). These are often the mothers (Cawley & Liu, 2012; Rosenkranz & Dzewaltowski, 2008; Walsh et al., 2015). For this study, we defined snacks as “all foods, healthy and unhealthy, consumed in between regular meals,” based on definitions used in previous studies (Duffey, Rivera, & Popkin, 2014; Garriguet, 2007; Hartmann, Siegrist, & Van Der Horst, 2013; Mercille, Receveur, & Macaulay, 2010; Ovaskainen, Tapanainen, & Pakkala, 2010).

Value conflicts were examined using food and motivation diaries and semi‐structured interviews. These two different qualitative data collection methods allow for within‐method triangulation, to enhance the validity of the results (Denzin, 2017; Thurmond, 2001). A grounded theory approach was used for data collection (Dew, 2007; Harris et al., 2009). Information on actual snack giving in the home environment and underlying considerations and value conflicts was collected with diaries, for the duration of 13 consecutive days, including two weekends, in January 2017. Within 2 weeks after completion of the diaries, semi‐structured interviews with the participants were conducted, as previously described by Turner (2010) and Creswell (2014). These interviews were held by telephone to gain additional information on value conflicts in snack giving, so not only focussing on the 2 weeks of keeping the diary but also when occurring in other situations. The designs of the diary research and the semi‐structured interviews were piloted with three mothers of young children not involved in the final study. Only minor changes to the diary study were made; according to this pilot study, the design of the interviews remained unchanged, except for replacement of the term “value conflicts” by the simpler term “difficult moments.”

### 2.2 Recruitment and selection of participants

To recruit participants, social media, posters at schools and day‐care centres in the Netherlands, and snowball sampling (Zarantonello & Luomala, 2011) were used. Potential participants (*n* = 180) completed a questionnaire on demographics to select them according to the criteria set for the target group. These criteria included having at least one child in the age group 2–7 years, having the intention to keep the diary for the full 2 weeks and being willing to have an interview at the end of the study. Another criterion was that the child did not have a severe food allergy or suffered from chronic diseases (e.g., diabetes). Ten respondents did not meet the inclusion criteria, and a disproportionate number of mothers of children aged 2–3 years responded. Therefore, in total, 41 respondents were excluded from the study, and 139 mothers were selected to participate. These 139 participants were purposively included (Draper & Swift, 2011; Harris et al., 2009) to vary regarding the order of the child in the household, age group of the child, and educational level of the mother. Mothers were divided into two groups according to their educational attainment. The group of higher educated mothers included mothers with a bachelor's degree or higher, the lower educated group mothers with a degree lower than the bachelor's.

Before the study started, participants received a letter explaining the duration and set‐up of the research. This letter also explicitly stated that the results would be handled anonymously, and all the personal data would have kept confidential following the rules of the data management plan of Wageningen University. Mothers had the possibility to withdraw from participation whenever they wanted. Each participant received a €40 gift voucher after keeping the diary and finishing the interview.

### 2.3 Diary research

Mothers reported every snack they gave themselves to their child in the morning, afternoon, and evening in an online, event‐based diary (Bolger, Davis, & Rafaeli, 2003). In addition, they reported their motivations, considerations, and satisfaction regarding these snack choices. Mothers were asked to fill in the diary for only one child. If they had more than one child in the target group of 2 to 7 years, the researcher instructed them for which child they should keep the diary. Data were collected using Qualtrics survey software (http://www.qualtrics.com, 2017) and could be accessed through any device with web access. Every morning, mothers received an email with the link to the diary. An additional email was sent only to those mothers who had not completed the diary by 9:00 pm in the evening. Mothers could decide themselves to report each snack immediately after the moment it was given or to report all snacks at the end of the day. For this reason, the mothers received a paper notebook to have the possibility to note the snacks and to complete the diary at their convenience later that day.

### 2.4 Semi‐structured interview

An interview scheme was developed to maintain consistency in interviewing. See, for details, Table 1.

**Table 1:** Interview guide

Interview guide
Review of the diary study
What were your experiences in keeping the diary on snack giving? Was filling in the diary of influence on your snack giving behavior? If no, could you explain? If yes, why? How? And in what extent? What did you experience as most striking in you snack giving behaviour in these 13 days of participation?
Value conflicts
What are for you difficult moments in providing snacks to your children? Why are these moments difficult for you? How do you deal with such moments? How do you feel about it? Can you give examples?

First, participants were asked about their experiences participating in the diary research, followed by what they experienced as most striking in their snack giving behaviour in the 13 days of participation. The main focus of the interviews was on the value conflicts participants experienced in providing snacks to their children in general, not only focused on the 13 days of the research but also on other days and in other situations; see Table 1. Two researchers carried out the interviews by telephone. Interviews ranged in duration from 5–30 min at a time convenient for the participant. Interviews were recorded digitally.

### 2.5 Data analysis

In total, 137 mothers completed the diaries and the subsequent semi‐structured interview. Data from the diaries, as well as transcribed data from the interviews, were imported in the software programme MaxQDA version 12. This programme was used to organize, code, and analyse the qualitative data. Diaries and interviews were coded separately; for each method, new code labels were developed. Coding was led by the first author and was done independently by both the first author and a second researcher. After coding the same 25 diaries, given code labels were compared, and differences were discussed and resolved to come to a set of code labels to be used for coding the remaining diaries to obtain consistency in coding. The same was done for coding of the interviews. Conventional content analysis was used to retrieve the categories of value conflicts from the diaries as well as from the interviews, as described by Hsieh and Shannon (2005). Codes with comparable meanings were merged to one value conflict. The type of value conflicts retrieved from the diaries, and the interviews were comparable, and therefore, six main value conflicts were defined based on the results from both the diaries and the interviews. The occurrence of these types of value conflicts was also analysed for the differences in mothers' educational attainment, first versus not‐first child, and the age groups of the children. Data saturation was reached, because after analysing a substantial set of diaries, no new value conflicts appeared; this was similar for the interviews.

## 3 RESULTS

### 3.1 Participant characteristics

Final analyses were based on a sample of 136 mothers, as one mother was excluded because she used another definition of snacks than instructed. On average, mothers were 33.9 years of age (*SD* = 4.4), 84% of them had a paid job working on average of 24.3 hr a week (*SD* = 6.5). Most mothers had two children (66%), 21% had three children, 8% had one child, and 5% had four children. Around half of the mothers (52%) had a lower educational level; the others (48%) had a higher educational level. Half of the mothers (50%) filled in the diary for their first child in the household, the others for their second or following child. The percentage of children in the three age groups 2–3 years, 4–5 years, and 6–7 years was respectively 35%, 33%, and 32%. In total, 134 out of the 136 interviews were analysed, as two interviews were not properly recorded.

### 3.2 Results for mothers' educational level, first versus not‐first child, and age groups of the children

During the 13 days of the diary research, mothers reported that they gave in total 2,415 snacks to their children and experienced value conflicts in 6% (*n* = 134) of these snack giving moments. These conflicts were experienced by 56% of the mothers (*n* = 76); the others reported not having experienced value conflicts. When value conflicts were mentioned, the explanation often included phrases like “I would prefer to give her..., but...,” “I know it is not the best choice, although...,” or “I gave him..., however....” Value conflicts mainly occurred when mothers gave unhealthy snacks like cookies, candy, crisps, or pie. During the interviews, most mothers (*n* = 114, 85%) mentioned experiencing one or more value conflicts when providing a snack to their young children.

From the diary as well as from the interviews, no differences appeared in the total number of value conflicts experienced between mothers with different educational levels. Diaries showed that mothers recording for first‐born children experienced value conflicts slightly more frequently compared with mothers recording for subsequent children in the household. This difference was especially present for more unhealthy snacks like cookies, pie, and crisps. From the interviews, this difference was not found.

I gave her the cookie she asked for. I preferred to give her a rice cracker, but she really wanted that cookie. I did not want to put energy into it and did not want the whining, so I gave her the cookie [ID131: higher educated, first child, 2–3 years, diary].

Mothers of younger children more often experienced value conflicts when providing a snack; this was seen in both the diary study and the interviews. The diaries revealed that almost half of the value conflicts were experienced by mothers of children aged 2–3 years, one‐third by mothers of children aged 4–5 years, and only one‐fifth by mothers of children aged 6–7 years. From the diaries, it appeared that 65% of the mothers who mentioned not experiencing value conflicts at all were mothers of children in the oldest age group of 6–7 years. Also from the interviews, we observed that mothers of older children experienced fewer value conflicts. Of all value conflicts mentioned in the interviews, 41% (*n* = 80) was mentioned by mothers of children aged 2–3 years, 33% (*n* = 65) by mothers of children aged 4–5 years, and 26% (*n* = 50) by mothers of children aged 6–7 years.

I gave her a piece of cake, because we had people with kids over for a birthday visit. However I preferred to give her fruit, so I am not totally happy with my choice [ID095: lower educated, first child, 2–3 years, diary].

### 3.3 Main value conflicts

This study elicited six main value conflicts experienced by mothers with young children, namely, conflicts between healthy and unhealthy snacks; conflicts between healthy and convenient snacks; conflicts related to providing snacks just before dinner; conflicts related to influence of others; conflicts when the child asks but the mother says “no”; and conflicts related to many unhealthy snacks at parties or visits. In the subsections below, these value conflicts will be explained and supported by verbatim quotes of the mothers. Table 2 provides an overview of the main value conflicts including illustrative quotes per value conflict.

**Table 2:** A selection of quotes that support the main value conflicts presented in the results

Main value conflicts with quotes from diary and interview
Conflicts of healthy versus unhealthy
I gave chocolate, but think it was better to give a healthier snack, because he also did not get his fruit today [ID048: lower educated, first child, 4–5 years, diary]; I did not provide a healthy snack, so I am not totally confident with my choice [ID112: higher educated, not‐first child, 6–7 years, diary]; She is a picky eater, so during main meals we have a lot of struggles and fights. I do not want to argue about the snacks, so if she only wants to eat banana as a fruit, it is okay. However, it feels difficult, I prefer a healthier option [ID005: lower educated, not‐first child, 2–3 years, interview].
Conflicts of healthy versus convenience
I think this (a chocolate) was not a good choice, I am a bit chubby myself, and therefore I want the kids to eat more healthily. However, with this snack I chose for convenience instead of health [ID117: lower educated, not‐first child, 6–7 years, diary]; I prefer to choose a healthy snack, like vegetables, but often I choose something else because of convenience [ID063: lower educated, first child, 2–3 years, interview]; That I sometimes choose for the more convenient snack is a fact, sometimes I am just too busy. At such moments, my children eat more candy or cookies than they normally do. Those are difficult moments for me [ID111: higher educated, not‐first child, 2–3 years, interview].
Conflicts related to providing a snack just before dinner
It was 30 minutes before dinner, giving a snack gives me doubts because it distracts them from eating dinner [ID116: higher educated, not‐first child, 2–3 years, diary]; At the end of the afternoon, it is a difficult moment. I need to start cooking in 30 minutes and then they start asking for a snack. Then I am in doubt, what shall I give? Because I do not want them to be satiated just before dinner [ID075: lower educated, first child, 6–7 years, interview]; Sometimes he asks for a snack just before dinner, I have difficulties to say no, but at such a moment I do, those are the difficult moments for me [ID109: lower educated, first child, 4–5 years, interview].
Conflicts related to influence of others
I prefer to give her a more nutritious snack, but when a friend is over to play she asks for something else. Because this is not happening every day I give in [ID061: higher educated, not‐first child, 4–5 years, diary]; Normally I prefer to give fresh fruit or dried fruit, however when others are around I become less strict [ID102, higher educated, not‐first child, 4–5 years, diary]; When others are around, it is more difficult for me to say no [ID028: lower educated, first child, 2–3 years, interview].
Conflicts related to child asks, mother says no
If they want a snack, and I do not agree, that is a difficult moment [ID014: higher educated, not‐first child, 6–7 years, interview]; It is difficult when they whine all the time, while I do not want to give it to them [ID051: higher educated, not‐first child, 4–5 years, interview]; If she asks for a snack I know she really likes and I say no, and she becomes really sad. Then it is difficult to keep saying no, I feel bad [ID063: lower educated, first child, 2–3 years, interview].
Conflicts related to unhealthy snacks at parties or visits
I prefer not to give her cake, but because it was a birthday I did. However, I do not really like this [ID113: lower educated, not‐first child, 2–3 years, diary]; He ate a small bowl of crisps with dip. I put some on the table because friends were visiting. So the children also wanted to eat it. I understand he wanted to have the same, however it did not feel good, because I want to protect my child from eating unhealthy foods [ID012: lower educated, first child, 6–7 years, diary]; I experience difficult moments if I go for a visit with my child and he is offered something else, while I would prefer to give him fruit. He normally never gets candy [ID064: lower educated, not‐first child, 2–3 years, interview].

#### 3.3.1 Conflicts of healthy versus unhealthy

Almost all value conflicts mentioned in the diaries and the interviews related to health. Especially, the discrepancy between healthy and (more) unhealthy snacking was a reason for experiencing value conflicts. For example, mothers preferred to give a healthy snack but ended up with a more unhealthy choice.

I intended to give some fruit, but gave an ice cream because she asked for it. There is nothing wrong with ice cream, however I think giving fruit is more important [ID053: lower educated, not‐first child, 2–3 years, diary];

I do not want to be a ‘hysterical' mother who never gives the nice snacks. However, I recognize that it feels more difficult for me if I provide them with a cookie with chocolate of which I know it contains loads of sugar. . . Then I always think, I wish I did not give it to them. However, I also think they are allowed to get something nice every now and then. So then I am in conflict with myself [ID113: lower educated, not‐first child, 2–3 years, interview].

#### 3.3.2 Conflicts of healthy versus convenience

When mothers preferred convenience above a healthier choice, they also experienced value conflicts. They cannot make another choice because they were too busy, and later, they felt guilty about the choice they made. This value conflict was mentioned both in the diaries and the interviews.

I gave ready to eat fruit puree, it is a convenient way of giving fruit, I was busy baking cake. However, I prefer to give “real” fruit [ID076: higher educated, first child, 4–5 years, diary];

Sometimes I want to give something healthy like fruit, but I am busy too. So I give something else instead, so I choose a more convenient option, in the end it feels bad [ID120: higher educated, first child, 4–5 years, interview].

#### 3.3.3 Conflicts related to providing a snack just before dinner

Mothers mentioned in the diaries and interviews that the time of the day is a reason for experiencing value conflicts. To illustrate, just before dinner, children become hungry and often ask for a snack, a situation many mothers experience as difficult. Mothers do not want their children to be hungry, but they also want their children to eat their dinner.

I gave a cookie, however I am not totally happy with the choice because it was just before dinner and she does not eat dinner that well. However, I was busy cooking dinner [ID045: lower educated, first child, 4–5 years, diary];

A difficult moment is just before dinner, if they start whining for a snack. At such a moment I sometimes think, hmmm I just give it to them [ID067: lower educated, not‐first child, 6–7 years, interview].

As a solution for this, mothers mentioned they try to provide their children healthier or less filling snack options, like vegetables or crackers.

The moment just before dinner is always difficult. Dinner is never ready in time. You have to pick up the children from day‐care or from their friends and you arrive home late. Cooking dinner always takes too long and they start whining for snacks. Sometimes I give in and sometimes I do not. However, I do not provide candy; they will get a carrot, a tomato or a bread stick. I do this because otherwise I know for sure they will not eat their dinner [ID121: higher educated, not‐first child, 4–5 years, interview].

#### 3.3.4 Conflicts related to influence of others

Mothers also frequently mentioned in the diaries that they would have preferred to give a healthy choice but changed their mind because of the influence of others.

We had children over to play. In that case, I always give candy. My experience is that most children are used to that and ask for it themselves. I do not want that other children do not like to come to play at our place because they do not get any candy [ID014: higher educated, first child, 6–7 years, diary].

In the interviews, it was also mentioned that when other children are around, mothers sometimes experienced value conflicts. They mentioned that they provide more snacks and that the snacks are unhealthier, like candy, cookies, or crisps.

If other kids come over to play I give candy or a cookie instead of something healthy more often. I try not to do so, but in those cases I feel a kind of pressure to please the kids [ID018: higher educated, not‐first child, 2–3 years, interview].

Another reason to provide more unhealthy snacks is that mothers do not want other children to judge their children based on the snacks they provide.

When kids come over to play, it is sometimes difficult. I give more unhealthy snacks than I normally do. I would feel bad if other children said to my children: “At your place we never get a nice snack” so eh. . . that my children will be judged because I do not give the nice snacks. . . Yes, then I would feel bad, so I try to find a balance in what I give [ID108: lower educated, not‐first child, 4–5 years, interview].

Another value conflict frequently mentioned in the interviews is when the child gets a snack from someone else, especially when the snack is not that healthy.

I experience difficulties if other people give a snack to my child, especially snacks I would not choose myself. For example a birthday treat, which only contained candy! I think that is not an appropriate snack to give [ID075: lower educated, first child, 6–7 years, interview].

#### 3.3.5 Conflicts related to child asks, mother says no

Mothers mentioned during the interviews that they experienced value conflicts when their child wanted to have a particular snack, but they did not agree with it. They mentioned that their child started to whine or even cried or screamed to try to get the particular snack. As the mothers did not want to capitulate, they said “no” but often felt bad and sometimes guilty, making these situations a value conflict in snack giving.

When they are asking and whining over and over again for that specific snack, and you are busy yourself. . . Then sometimes you just give that snack. Yes, to stop the whining. For me that is a very difficult moment [ID098: higher educated, not‐first child, 2–3 year, interview].

In the diaries, this conflict was not reported as only the considerations on the actual snacks given were part of the diary research.

#### 3.3.6 Conflicts related to unhealthy snacks at parties or visits

In the interviews, the most frequently mentioned value conflict was when there was a party or a visit from others. Overall, mothers experienced these value conflicts because many snacks were available for their children to take, others were around, and there was a convivial atmosphere.

When there is a party, there are a lot of snacks available on the table. My children want to take a snack almost every second, and then I have trouble in saying no. When other children are around and they are allowed to take the snacks, I allow my children to do so too, however this does not feel okay [ID003: higher educated, not‐first child, 6–7 years, interview].

In the diaries, this value conflict was also mentioned; however, not all mothers had a party or a visit during these 13 days.

Finally, mothers also made remarks in their diaries and interviews when they were very satisfied or happy about their choice. This was especially the case when a healthy choice was in line with the preference of their child.

My child wanted a tangerine. I felt proud of her, because she asked for a healthy snack and not for a candy [ID026: higher educated, not‐first child, 6–7 years].

## 4 DISCUSSION

This study described the value conflicts mothers experienced while providing a snack to their young children. Mothers in this study experienced most value conflicts when they provided snacks they perceived as unhealthy, like cookies, candy, crisps, and pie. This is in line with the research of Pescud and Pettigrew (2014), who reported that guilt is an emotion that parents increasingly experience when providing unhealthier or too much foods to their children.

No differences in the number of value conflicts appear between mothers with different educational levels. This result was unexpected, as previous analyses on this dataset revealed that higher educated mothers showed more health‐conscious snack giving behaviour compared with lower educated mothers (Damen, Luning, et al., 2019). Moreover, others (Bargiota, Delizona, Tsitouras, & Koukoulis, 2013; Durão et al., 2017; Emmett & Jones, 2015; Gevers et al., 2016; Saldiva et al., 2014; van Ansem, van Lenthe, Schrijvers, Rodenburg, & van de Mheen, 2014; Vilela et al., 2015) also found higher educated mothers to be more health‐conscious compared with lower educated mothers. Because most value conflicts in the current study are health related, it was expected that when mothers were more health‐conscious, they would also experience value conflicts more often.

Mothers of younger children more often experienced value conflicts when providing a snack than mothers of older children. Parents feel that healthy‐eating habits should start at an early age, preferably when children become toddlers (Nepper & Chai, 2016). Moreover, at this stage, food neophobia and picky eating behaviour usually start (Dovey, Staples, Gibson, & Halford, 2008). Russell, Worsley, and Campbell (2015) found that mothers of children who are picky eaters experience more negative emotions and use more often nonresponsive feeding practices such as using snacks as a reward and give pressure to their child to eat certain foods. Various studies (e.g., French, Epstein, Jeffery, Blundell, & Wardle, 2012; Savage et al., 2018; Stifter, Anzman‐Frasca, Birch, & Voegtline, 2011) showed that using more responsive feeding practices to young children, so be responsive to children's cues of hunger and fullness and support children's self‐regulation in eating (Russell et al., 2015), helps in preventing obesity risk. Carrigan, Szmigin, and Leek (2006) found that when children were very young, preparing homemade food was more important for mothers. They perceived it as their responsibility to provide their children a diet that would enable them to grow up healthy. In accordance, Carnell et al. (2011) reported that some mothers became more flexible in feeding their children when their children became older. The higher responsibility that mothers may feel when providing food to their young children might explain why in the current study mothers of younger children experienced value conflicts more often.

Mothers of first‐born children reported more value conflicts in the diary study but not in the interviews. Previous research showed that mothers of first‐born children are more careful regarding the healthiness of the snacks they provide (Damen, Luning, et al., 2019). Also, Brekke, van Odijk, and Ludvigsson (2007) and Smith, Emmett, Newby, and Northstone (2011) showed that mothers of first children behave more health‐conscious compared with mothers of subsequent children. Although the purposive sampling of the participants in this research allow for comparison of mothers with first‐born and not first‐born children, mothers' educational attainment, and age groups of the children, it does limit the interpretation of the results to a more general population.

The mothers frequently mentioned a value conflict between healthy and unhealthy snacks, both in the diaries as well as in the interviews. Often, mothers preferred to give a healthy snack but ended up with an unhealthier one and therefore felt bad. Also Hayter et al. (2015) reported that parents often mention differences in what they would like to feed their children and what they actually provide. Luomala et al. (2004) concluded that one of the most common value conflicts in food choice is the conflict between health and indulgence. In their study, respondents mentioned they knew about the importance of healthy eating and mentioned self‐indulgence as an essential part of their lives (Luomala et al., 2004). Okada (2005) concluded that people feel guiltier about eating indulgent hedonic foods, such as candies, than they feel for healthier snacks, like fruits or vegetables because consuming hedonic foods gives need for justification. Also in the current study, mothers experienced more value conflicts while providing unhealthier snacks.

Another value conflict observed in both the diaries and interviews is the one between health and convenience. When mothers preferred to provide a healthy snack, but they are too busy, they provided their children a more convenient snack. Because such a snack was usually not perceived as healthy, they felt guilty and experienced value conflicts. Various studies underpinned the role of time constraints in providing healthy foods to children; however, these studies mainly focused on main meals rather than snack foods. Nepper and Chai (2016), who studied parents' barriers in healthy eating among school‐aged children, reported that parents have trouble in providing healthy foods during the main meals, because they are busy and strapped for time. Hayter et al. (2015) investigated perceptions of low‐income parents in the United Kingdom about feeding their preschool children. They found that parents express conflicts between what they would like to provide their children as a food and what they could provide due to time constraints. In a study by Walsh et al. (2015) about how mothers make food choices for their preschool children, mothers frequently mentioned a lack of time to prepare healthy meals. Likewise, Damen et al. (Damen et al., 2019) found that convenience is an important value for Dutch mothers and that they sometimes lack time to prepare healthy snacks for their children. Pettigrew and Roberts (2007) reported that mothers feel guilty about their children's health and doubt about being a good mother, because of the convenient choices they made for main meals, which is in line with the value conflicts observed in the current study.

Just before dinner, mothers perceive difficulties when providing a snack; this was reported in both the diaries and in the interviews. Holsten et al. (2012) reported that 11‐ to 14‐year‐old children described that parents affected their food choice by rules they set. One of the rules mentioned is not to eat something just before dinner because their parents expect them to eat their dinner.

The influence of other persons, like grandparents, spouses, or friends, often triggered mothers' value conflicts. Especially when others provided the children an unhealthy snack, mothers experienced value conflicts. Likewise, Walsh et al. (2015) described that mothers experience challenges in food choice when extended family members, with different views on giving snacks, are present. Similarly, Boak et al. (2016) concluded that the presence of others, like grandparents, extended family, or friends, could have an influence on foods mothers choose for their infants. Pettigrew and Roberts (2007) reported that mothers feel undermined by their spouse or other direct family in their ability to control the quality of their child's diets. Herman, Malhotra, Wright, Fisher, and Whitaker (2012) described the influencing role of grandparents in the provision of snacks as being difficult for mothers. In the current study, mothers also experienced value conflicts in providing snacks to their children when other children came over to play. Also, Walsh et al. (2015) described that mothers struggle with food choice for their own children when other children are around. Mothers in the current study mentioned that they provided more and unhealthier snacks, because they did not want their children to be judged by other children on the type of snacks they provided.

In addition, the interviews showed that mothers experienced value conflicts when their child wanted to have a particular snack, and the mother says “no,” especially when their child started whining. Nepper and Chai (2016) reported that parents have strategies to deal with food requests of their children and feel conflicts between giving what their child asks for and what they prefer to give. In a focus group with mothers of children aged 1 to 12 years on child feeding in general (Pettigrew & Roberts, 2007), mothers described the conflict between what the child asks for and what they would like to provide. Herman et al. (2012) described that low‐income mothers have difficulties to say “no” when their children request snack foods and that they feel bad and frustrated when they give in.

Another value conflict mothers frequently mentioned in the interviews occurred when there was a party when others visited. Levinson, Mack, and Reinhardt (1992) stated that celebrations and cultural festivities expose values that can remain hidden in everyday consumer behaviour. During parties or visits of others, most of the available snacks were unhealthy. Therefore, mothers struggled with limiting the amount of unhealthy snacks taken by their children without getting complaints. Similarly, Pescud and Pettigrew (2014) reported that parents experience feelings of guilt associated with the provision of large quantities of unhealthy foods at special occasions such as parties.

The current study elicited six main value conflicts mothers experience when providing snacks to their children. These are conflicts between healthy and unhealthy snacks; conflicts between healthy and more convenient snacks; conflicts related to providing snacks just before dinner; conflicts related to the influence of others; conflicts related to the child asks but the mother says “no”; and conflicts related to many unhealthy snacks at parties or visits. To the best of our knowledge, no studies have been conducted specifically focussing on value conflicts in providing snacks to young children before. Previous studies mainly focused on main meals or on snack eating behaviour of older children. Moreover, the study is unique because it used two types of methods: measuring value conflicts in daily life using diaries and semi‐structured interviews of a relatively large number of mothers. The understanding that was gained about these value conflicts of mothers of young children can be useful for interventions to promote more healthy eating behaviour among children. In addition, the results can be of support in the design of (new) snack products, which can help to reduce the value conflicts experienced by mothers of young children. For example, snacks could be designed, which are more healthy alternatives of existing unhealthy snacks or snacks that are both convenient and healthy.

## CONFLICTS OF INTEREST

The authors declare that they have no conflicts of interest.

## CONTRIBUTIONS

The authors' responsibilities were as follows: FD and BS designed the study; FD conducted the research, analysed the data, and drafted the manuscript; BS and PL provided conceptual and methodological guidance; all authors critically revised and approved the manuscript.
